# Effect of Fractional Carbon Dioxide vs Sham Laser on Sexual Function in Survivors of Breast Cancer Receiving Aromatase Inhibitors for Genitourinary Syndrome of Menopause: The LIGHT Randomized Clinical Trial

**DOI:** 10.1001/jamanetworkopen.2022.55697

**Published:** 2023-02-10

**Authors:** Eduard Mension, Inmaculada Alonso, Sònia Anglès-Acedo, Cristina Ros, Jorge Otero, Álvaro Villarino, Ramon Farré, Adela Saco, Naiara Vega, Natalia Castrejón, Jaume Ordi, Natalia Rakislova, Marta Tortajada, Isabel Matas, Sílvia Gómez, Laura Ribera, Camil Castelo-Branco

**Affiliations:** 1 Clinic Institute of Gynecology, Obstetrics and Neonatology, Faculty of Medicine-University of Barcelona, Hospital Clínic of Barcelona, Barcelona, Spain; 2 Gynecology, Obstetrics and Neonatology Service, Hospital Joan XXIII, Tarragona, Spain; 3 Unit of Biophysics and Bioengineering, Faculty of Medicine, University of Barcelona, Barcelona, Spain; 4 CIBER de enfermedades Respiratorias, Madrid, Spain; 5 Department of Pathology, Hospital Clínic of Barcelona, University of Barcelona, Barcelona, Spain; 6 Barcelona Institute for Global Health, Hospital Clínic, Universitat de Barcelona, Barcelona, Spain; 7 Institut d´Investigacions Biomèdiques August Pi i Sunyer, Barcelona, Spain

## Abstract

**Importance:**

Survivors of breast cancer present more severe symptoms of genitourinary syndrome of menopause (GSM) than patients without history of breast cancer. Recently, new treatments, such as vaginal laser therapy, have appeared, but evidence of their efficacy remains scarce.

**Objective:**

To assess the safety and efficacy of carbon dioxide (CO_2_) vs sham vaginal laser therapy after 6 months of follow-up in survivors of breast cancer with GSM receiving aromatase inhibitors.

**Design, Setting, and Participants:**

This prospective double-blind sham-controlled randomized clinical trial with two parallel study groups was performed during October 2020 to March 2022 in a tertiary referral hospital. Survivors of breast cancer using aromatase inhibitors were assessed for eligibility, and eligible patients were randomized into the 2 treatment groups. Follow-up was conducted at 6 months. Data were analyzed in July 2022.

**Interventions:**

All patients from both groups were instructed to use the first-line treatment (FLT) based on nonhormonal moisturizers and vaginal vibrator stimulation. Patients for each group were allocated to 5 monthly sessions of fractional CO_2_ laser therapy (CLT) or sham laser therapy (SLT).

**Main Outcomes and Measures:**

The primary outcome was sexual function, evaluated through Female Sexual Function Index (FSFI) score. Other subjective measures of efficacy included a visual analog scale of dyspareunia, vaginal pH, a Vaginal Health Index, quality of life (assessed via Short-Form 12), and body image (assessed with the Spanish Body Image Scale). Objective measures of efficacy included vaginal maturation index, vaginal epithelial elasticity (measured in Pascals) and vaginal epithelial thickness (measured in millimeters). Measures were assessed before and after the intervention. Tolerance (measured on a Likert scale), adverse effects, and estradiol levels were recorded.

**Results:**

Among 211 survivors of breast cancer assessed, 84 women were deemed eligible and 72 women (mean [SD] age, 52.6 [8.3] years) were randomized to CLT (35 participants) or SLT (37 participants) and analyzed. There were no statistically significant differences between groups at baseline. At 6 months, both groups showed improvement in FSFI (mean [SD] score at baseline vs 6 months: CLT, 14.8 [8.8] points vs 20.0 [9.5] points; SLT, 15.6 [7.0] points vs 23.5 [6.5] points), but there was no significant difference between CLT and SLT groups in the improvement of sexual function evaluated through the FSFI test overall (mean [SD] difference, 5.2 [1.5] points vs 7.9 [1.2] points; *P* = .15) or after excluding women who were not sexually active (mean [SD] difference, 2.9 [1.4] points vs 5.5 [1.1] points; *P* = .15). There were also no differences between improvement of the 2 groups at 6 months of follow-up in the other assessed subjective outcomes, including dyspareunia (mean [SD] difference, −4.3 [3.4] vs −4.5 [2.3]; *P* = .73), Vaginal Health Index (mean [SD] difference, 3.3 [4.1] vs 5.0 [4.5]; *P* = .17), body image (mean [SD] difference, −3.7 [4.5] vs −2.7 [4.8]; *P* = .35), and quality of life (mean [SD] difference, −0.3 [3.6] vs −0.7 [3.2]; *P* = .39). Similarly, there were no differences in improvements in objective outcomes, including vaginal pH (mean [SD] difference, −0.6 [0.9] vs −0.8 [1.2]; *P* = .29), vaginal maturation index (mean [SD] difference, 10.2 [17.4] vs 14.4 [17.1]; *P* = .15), vaginal epithelial thickness (mean [SD] difference, 0.021 [0.014] mm vs 0.013 [0.012] mm; *P* = .30), vaginal epithelial elasticity (mean [SD] difference, −1373 [3197] Pascals vs −2103 [3771] Pascals; *P* = .64). There were significant improvements in the overall analysis regardless of group in many outcomes. The 2 interventions were well tolerated, but tolerance was significantly lower in the CLT group than the SLT group (mean [SD] Likert scale score, 3.3 [1.3] vs 4.1 [1.0]; *P* = .007). No differences were observed in complications or serum estradiol levels.

**Conclusions and Relevance:**

In this randomized clinical trial, vaginal laser treatment was found to be safe after 6 months of follow-up, but no statistically significant differences in efficacy were observed between CLT and SLT.

**Trial Registration:**

ClinicalTrials.gov identifier: NCT04619485

## Key Points

**Question:**

Is vaginal laser treatment safe and effective for genitourinary syndrome of menopause in survivors of breast cancer receiving aromatase inhibitors?

**Findings:**

In this randomized clinical trial with 84 participants in 2 parallel study groups, both groups received a first-line therapy based on nonhormonal moisturizers and vaginal vibrator stimulation, and participants were randomized to receive 5 weekly sessions of fractional carbon dioxide laser therapy or sham laser therapy. No differences were observed between groups in safety or efficacy outcomes at the 6-month follow-up.

**Meaning:**

These findings suggest that although vaginal laser treatment was safe, it was not more effective than first-line therapy with placebo treatment in survivors of breast cancer receiving aromatase inhibitors.

## Teaser

This randomized clinical trial assesses the safety and efficacy of carbon dioxide (CO_2_) vs sham vaginal laser treatment after 6 months of follow-up in survivors of breast cancer receiving aromatase inhibitors with genitourinary syndrome of menopause.

## Introduction

During the last decades, dysfunction of sexual and vaginal health, including genitourinary syndrome of menopause (GSM), has remained underdiagnosed and undertreated in survivors of breast cancer.^1,2^ These symptoms are usually worse among survivors of breast cancer compared with women without history of cancer, due to the antiestrogenic effects of chemotherapy, tamoxifen, and aromatase inhibitors.^3^ In addition, estrogen-based standard treatment for GSM remains controversial in this subset of patients.^4^

In the last few years, new therapeutic approaches have been designed to relieve GSM symptoms, and vaginal laser therapy is one of the trending options.^5^ Although most studies conclude that vaginal laser therapy is a safe option,^6,7^ safety outcomes are underreported in most studies.^8^ In a 2022 systematic review,^8^ vaginal laser treatment was associated with improved subjective outcomes, such as the dyspareunia (assessed via visual analog scale [VAS]), the Female Sexual Function Index (FSFI), and the Vaginal Health Index (VHI), in the short term; however, most of the assessed studies were single-group before-and-after trials with evidence of low to moderate quality.^8^ There is also controversy on the results of objective outcomes, as some studies^9,10^ have shown an increase of the epithelial vaginal layer, whereas other studies^11,12^ have found no differences between sham and real laser groups. Thus, there is need for sham-controlled randomized clinical trials (RCTs). The aim of this study was to assess the safety and efficacy of carbon dioxide (CO_2_) vaginal laser therapy (CLT) compared with sham laser therapy (SLT) after 6 months of follow-up in survivors of breast cancer with GSM receiving aromatase inhibitors.

## Methods

This RCT was approved by the institutional review board of the Hospital Clinic of Barcelona, Spain. This study adheres to the European Union Law of Data Protection and was conducted ethically in accordance with the Declaration of Helsinki. Written informed consent was obtained from all participants. This study followed the Consolidated Standards of Reporting Trials (CONSORT) reporting guideline for RCTs.

### Study Design

In this prospective, double-blind, sham-controlled, RCT with 2 parallel study groups, both groups received first-line therapy (FLT) based on nonhormonal moisturizers and a vaginal vibrator stimulation, plus 5 monthly sessions of laser treatment with 2 groups, the first receiving fractional CLT and the second receiving SLT. The trial protocol and statistical analysis plan are presented in Supplement 1.

### Participants

The study was conducted in the Hospital Clinic of Barcelona, Spain. The inclusion criteria were survivors of breast cancer aged 30 years and older receiving aromatase inhibitors (for ≥6 months); menopause, signs or symptoms of GSM with dyspareunia, and vaginal pH of 5 or greater; and self-reported willingness to be sexually active. The exclusion criteria included use of vaginal moisturizers or lubricants in the last 30 days; vaginal hormonal treatment in the last 6 months; use of radiofrequency, laser treatment, hyaluronic acid, or lipofilling in the vagina in the last 2 years; ospemifene treatment; intraepithelial neoplasm of cervix, vagina, or vulva; active genital tract infection; prior treatment for genital cancer; organ prolapse stage II or greater; and positive test results for human papillomavirus. Recruitment began in October 2020 and finished in September 2021. Ethnicity was self-reported by patients and assessed to describe the cohort.

### Sample Size Calculation and Randomization

Considering the FSFI score as the primary outcome, a sample size of 33 women was calculated for each group, accepting an α risk of 0.05 and a β risk of less than 0.1 in a bilateral contrast. The common SD was considered to be 5 points, and the minimum expected effect size was 4 points.^13,14^ Assuming a loss to follow-up of 15%, the calculated sample size was 78 patients.

Participants were equally assigned by 1:1 block randomization to either CLT or SLT using Stata software version 15.1 (StataCorp). The block sizes were 8. Allocation concealment was performed using a protected personal code folder on the hospital intranet. Access to the randomization folder was limited to an authorized collaborator physician who had no other involvement in the study.

### Interventions

At the first visit, patients completed all the questionnaires. Additionally, participants underwent a vaginal examination to evaluate the genital tract and collect samples for analysis.

#### First-line Therapy

All patients from both groups were instructed to use the FLT, which was supplied to every participant during the study. This therapy included a hormone-free moisturizer containing hyaluronic acid (Cerviron; CumLaude Lab) to be used every 3 days, a daily external vaginal hormone-free moisturizer (Lubripiu; CumLaude Lab), and a vaginal vibrator (Meditinum; BCNatal) to be used 2 times per week for 5 to 10 minutes each with the help of intimate lubricant (Mucus). A personal calendar was given to each patient in which they recorded every use of the moisturizer, the vaginal vibrator, and each sexual relation practiced. Additionally, specialized sexual assessment was also offered as an optional visit based on the PLISSIT (Permission, Limited Information, Specific Suggestions, and Intensive Therapy) model.^15^

#### Preparation for the Procedure

The patients were scheduled between 4 to 6 weeks after the first visit. They were instructed to avoid intercourse and use an internal vaginal ovule moisturizer daily 5 days prior and 5 days after the laser session and use topic lidocaine cream 1 hour before the laser session.

#### Laser Treatment

All patients underwent 5 sessions 1 month apart from the vaginal laser treatment. The treatment was performed by a professional blinded to the treatment group.

CLT was performed using the fractional microablative CO_2_ laser system, SmartXide2 V^2^LR, MonaLisa Touch (DEKA Laser) at standard settings (40W power, 1000 μs dwell time, 1000 μm dot spacing, SmartStack 2 on double pulse emission mode), with a delivery fluence of 5.37 J/cm^2^. SLT was performed at minimal energy settings to avoid any tissue effect (0.0 W power, 100 μs dwell time, 2000 μm dot spacing, SmartStack 1 on SmartPulse emission mode), delivering no energy (0 J/cm^2^).

All patients reporting symptoms suspicious of vulvovaginal candidiasis or urinary tract infection prior to the laser session were treated accordingly. Sessions were rescheduled until treatment was completed.

The first step of the procedure involved removal of the external anesthetic cream with a dry gauze. Then, using an exploration speculum, a new dry gauze was inserted into the vaginal canal to remove all residual vaginal moisture. Next, the laser probe was inserted into the vagina without lubrication. A 360° laser probe was used as the first option, but when the diameter was too large, a 90° probe was used. The laser pulses were delivered to treat the entire circumference and length of the vagina from the apex to the introitus. Patients had no visual stimuli since opaque glasses were used; neither was there olfactory stimulus from smoke plume due to the use of an aspirator during the procedure. Auditory stimuli from the laser and aspirator were set to be equal between groups.

### Masking

The laser parameters were manually entered by an assistant, and the gynecologist and participants were masked. Only the assistant had access to the randomization folder. Participants could not guess in which group they were allocated, as they were informed that the laser treatments might not produce any discomfort.

### Outcomes

Outcomes were assessed on the first visit prior to the initiation of any treatment and 6 months later (ie, 1 month after the fifth laser session). The primary outcome was sexual function, measured using the FSFI. Secondary outcomes included both objective and subjective measures.

#### Primary Outcome

The FSFI is a generic sexual questionnaire that has been validated for survivors of cancer.^15,16^ It assesses 6 sexual dimensions (desire, arousal, lubrication, orgasm, satisfaction, and pain). Global sexual function results in a score ranging from 2 to 36 points, with a higher score indicating better sexual function. A cutoff 26.55 points or lower identifies women at risk of female sexual dysfunction.^17,18^

#### Subjective Secondary Outcomes

##### Dyspareunia

The intensity of dyspareunia was assessed in all patients (sexually active and inactive) at the baseline visit according to their last vaginal sexual activities. Patients were asked to complete a VAS ranging from 0 to 10, with higher score indicating worse dyspareunia.

##### Body Image 

Body image was assessed using the Spanish Body Image Scale (S-BIS), a Spanish-language validated questionnaire assessing affective, behavioral, and cognitive body image dimensions in 10 items. The total score is the sum of all the items (range, 0-30), with higher scores indicating more concern regarding body image.^19^

##### Quality of Life 

Quality of life was measured using the Short-Form 12 (SF-12) test, which consists of a total of 12 items in 8 subdimensions on physical functioning. Scores range from 0 to 100, with higher scores indicating better quality of life.^20^

##### Vaginal Health Index

The VHI subjectively assesses the elasticity of the vagina, the amount of discharge, the integrity of the epithelium, and humidity, along with pH as the only objective criteria. The results range from 5 to 21, and scores of 15 or lower indicate vulvovaginal atrophy.^21^

#### Objective Secondary Outcomes

##### Vaginal pH

To assess vaginal pH, a piece of litmus paper is placed on the lateral vaginal wall until moistened. A pH of 4.6 or higher indicates vaginal atrophy.^22^

##### Vaginal Maturation Index

Cytological samples were collected to assess Vaginal Maturation Index and were assessed by gynecological cytologists blinded to the randomization group and sample sequence (before or after treatment). The relative proportion of parabasal, intermediate, and superficial vaginal epithelial cells was assessed.^23^ Vaginal Maturation Index scores range from 5 to 25, with higher scores indicating better vaginal health status.

##### Vaginal Epithelium Thickness

To assesses vaginal epithelium thickness (VET), 2 full-thickness vaginal mucosal samples taken from the right vaginal wall 2 to 3 cm above the introitus were obtained using Tischler biopsy forceps after local lidocaine infiltration. One of the specimens was fixed in formalin and routinely embedded in paraffin for histological evaluation, and 4-μm sections were stained with hematoxylin and eosin and digitized using a IntelliSite Ultra-Fast Scanner (Philips). The slides were evaluated and measured by a gynecologic pathologist. VET was microscopically evaluated by calculating the mean of the 3 areas showing the maximum VET and the 3 areas demonstrating the minimum VET in hematoxylin and eosin–stained tissue samples.

##### Vaginal Epithelium Elasticity

The second biopsy sample was used for evaluation of vaginal epithelium elasticity (VEE). VEE measurements were conducted using a customized Atomic Force Microscope (TE2000) equipped with a V-shape cantilever (0.13 N/m) ending with a polystyrene bead spherically shaped with a radius of 4.5 μm (Novascan). Micromechanics were examined by indenting the sample with the bead while recording the force applied, as described in Alcaraz et al.^24^ The biophysics investigators were blinded to the randomization group and sample sequence.

##### Adverse Effects and Tolerance

Immediate adverse effects (AEs), such as bleeding or laceration, were evaluated after every laser session. Late AEs, such as vaginal itching or urinary tract infections, were evaluated in later visits. All AEs were recorded and graded according to the National Cancer Institute Common Terminology Criteria for Adverse Events version 5.0.^25^ Tolerance to the intervention was assessed using a Likert scale, with scores ranging from 1 to 5, with higher scores indicating more tolerability.

### Statistical Analysis

Statistical analyses were performed with Stata software version 15.1 in July 2022. Normal distribution of the sample was evaluated using the Shapiro-Wilk test. Analyses of the main outcome (FSFI) and secondary outcomes were performed. Continuous variables were compared using the independent or paired-samples *t* test and presented as mean and SD. Contingency tables were assessed using the Fisher exact test. A 2-sided *P* < .05 was considered statistically significant.

## Results

A total of 211 women who had been treated for breast cancer were assessed for eligibility. Of these, 84 women were randomized into the 2 treatment groups and 72 participants (mean [SD] age, 52.3 [8.3] years) were analyzed, including 35 patients randomized to CLT and 37 patients randomized to SLT. The Figure shows the flowchart of the patients initially recruited in each arm and the women excluded with details of exclusion criteria. The demographic characteristics of the 2 study groups are shown in Table 1. No differences in any of the parameters were observed between groups.

**Figure.**
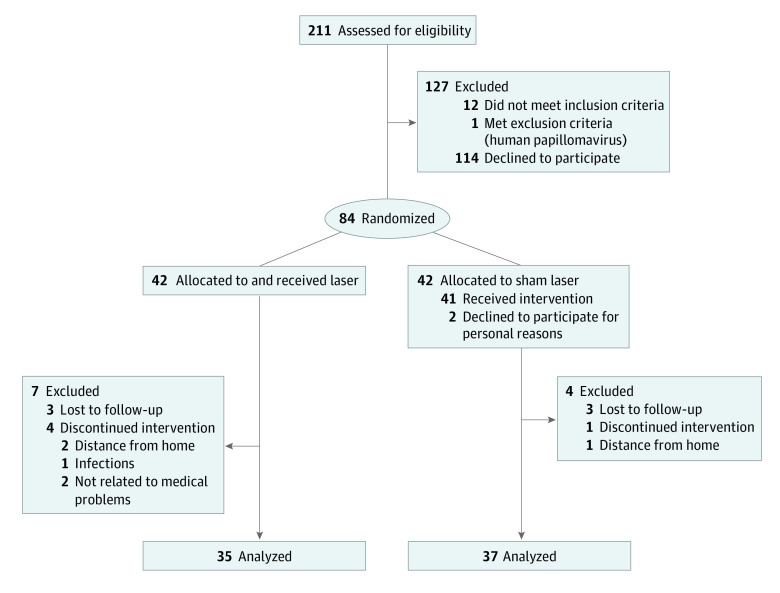
Participant Flowchart

**Table 1.** Demographic Characteristics of the Two Study Groups

Characteristic	Participants, No. (%)	
	Laser group (n = 35)	Sham laser group (n = 37)
Age, mean (SD), y		
At enrollment	51.3 (7.8)	53.7 (8.8)
At menopause	44.7 (6.7)	45.6 (5.8)
BMI, mean (SD)	23.9 (4.6)	24.9 (3.9)
Type of menopause		
Natural	9 (25.7)	17 (46.0)
Induced	26 (74.3)	20 (54.0)
Smokers	6 (17.2)	2 (5.4)
Race and ethnicity		
White	33 (94.3)	36 (97.3)
Latin	2 (5.7)	1 (2.7)
Parity (have children)	26 (76.5)	27 (73.0)
Mental health management		
Nonpharmacological	18 (51.4)	24 (64.9)
Pharmacological	17 (49.6)	13 (35.1)
Time since breast cancer diagnosis, mean (SD), y	3.5 (3.0)	4.8 (3.2)
Nodal status		
No metastases (pN0)	22 (62.9)	28 (77.8)
Metastatic lymph nodes (≥pN1)	13 (37.1)	8 (22.2)
Surgery		
Conservative surgery	18 (52.9)	17 (46.0)
Mastectomy		
No reconstruction	9 (26.5)	13 (35.1)
Reconstruction	5 (20.6)	7 (18.9)
Adjuvant therapy		
Hormone therapy	35 (100)	37 (100)
Radiation therapy	25 (71.4)	26 (70.3)
Chemotherapy	29 (82.9)	28 (75.7)
Initially sexually active	25 (71.4)	27 (73.0)

Abbreviation: BMI, body mass index (calculated as weight in kilograms divided by height in meters squared).

### Overall Outcomes Before and After Treatment

Table 2 shows the overall outcomes before and after treatment of all the patients included in the study. All 72 patients used the FLT, and the mean (SD) use of vaginal ovules was 9.5 (X.X) per month. The mean (SD) use of the vibrator was 5.6 (X.X) times per month. The mean (SD) monthly frequency of sexual activity was 2.7 (X.X) events. There was significant improvement in all the subjective and objective parameters at the 6-month follow-up, except in quality of life and VET.

**Table 2.** Overall Outcomes Before and After Treatment of All Patients Included in the Study

Outcome	Mean (SD)		Difference, mean (SD) [95% CI]	*P* value^a^
	Baseline (n = 72)	6-Month follow-up (n = 72)		
**Primary outcome: female Sexual Function Index score, points^b^**				
All women	15.2 (7.9)	21.8 (8.1)	6.4 (0.9) [4.7 to 8.3]	<.001
Sexually active women (n = 52)	18.9 (5.2)	23.2 (7.3)	4.3 (0.9) [2.7 to 6.3]	<.001
Subscores				
Desire	2.4 (1.0)	3.1 (1.1)	NA	NA
Arousal	3.6 (1.3)	4.2 (1.1)	NA	NA
Lubrication	3.3 (1.4)	3.8 (1.5)	NA	NA
Orgasm	3.8 (1.5)	4.4 (1.5)	NA	NA
Satisfaction	3.6 (1.9)	4.2 (1.7)	NA	NA
Pain	2.3 (1.7)	3.6 (1.8)	NA	NA
**Subjective outcomes**				
Dyspareunia (visual analog scale)^c^	7.6 (2.3)	3.1 (2.6)	−4.5 (3.8) [−4.9 to −3.4]	<.001
Vaginal health index^d^				
Overall	10.4 (3.1)	14.6 (3.6)	4.2 (0.5) [3.1 to 5.2]	<.001
Elasticity	2.4 (1.0)	3.1 (0.8)	NA	NA
Fluid secretion	2.0 (0.9)	3.0 (0.9)	NA	NA
Vaginal pH	1.2 (0.6)	1.5 (1.0)	NA	NA
Epithelial mucosa	2.6 (0.9)	3.5 (0.9)	NA	NA
Moisture	2.4 (1.0)	3.6 (1.0)	NA	NA
Body Image Scale-Spanish version^e^	11.1 (7.0)	7.9 (6.6)	−3.2 (0.5) [−4.1 to −1.8]	<.001
Short Form-12^f^	32.1 (2.5)	31.5 (3.1)	−0.6 (0.4) [−1.2 to 0.5]	.38
**Objective outcomes**				
Vaginal pH	7.8 (0.3)	7.1 (1.2)	−0.7 (0.1) [−0.9 to −0.4]	<.001
Vaginal Maturation Index^g^	5.9 (13.7)	18.4 (17.5)	12.5 (2.3) [8.2 to 17.4]	<.001
Vaginal biopsy thickness, mm	0.091 (0.061)	0.108 (0.045)	0.017 (0.001) [−0.003 to 0.033]	.10
Vaginal elasticity, Pascals	5095.1 (3232.9)	3492.8 (1605.7)	−1603.3 (610.4) [−2985 to −489]	.007
Serum estradiol, pg/mL	13.3 (37.7)	7.5 (11.4)	−5.8 (8.7) [−28.7 to 7.8]	.25

^a^ Calculated with *t* test.

^b^ Range, 2 to 36 points; lower scores indicate worse sexual dysfunction.

^c^ Assessed with a visual analog scale ranging from 0 to 10, with higher score indicating worse dyspareunia.

^d^ Range, 5 to 21; scores of 15 or lower indicate vulvovaginal atrophy.

^e^ Range, 0 to 30; higher scores indicate more concern regarding body image.

^f^ Range, 0 to 100; higher scores indicate better quality of life.

^g^ Range, 0-100; higher scores indicate better vaginal trophism.

### Primary Outcome

There was improvement of the primary sexual function after treatment evaluated through the FSFI test in the overall analysis, regardless of group. Overall, FSFI improved from a mean (SD) of 15.2 (7.2) points at baseline to 21.8 (8.1) points at the 6-month follow-up (*P* < .001). Excluding women who were not sexually active did not change the results (mean [SD] score: baseline, 18.9 [5.2] points; follow-up: 23.2 [7.3] points; *P* < .001).

At 6 months, both groups showed improvement in FSFI (mean [SD] score at baseline vs 6 months: CLT, 14.8 [8.8] points vs 20.0 [9.5] points; SLT, 15.6 [7.0] points vs 23.5 [6.5] points). However, there was no significant difference between CLT and SLT groups in the change in sexual function evaluated through the FSFI test at 6 months (mean [SD] difference, 5.2 [1.5] points vs 7.9 [1.2] points; *P* = .15). After excluding women who were not sexually active, there was still no significant difference (mean [SD] difference, 2.9 [1.4] points vs 5.5 [1.1] points; *P* = .15).

### Secondary Outcomes After Treatment by Randomized Groups

Results of the secondary outcomes evaluated before and after treatment in the 2 groups are shown in Table 3 and the eFigure in Supplement 2. None of the parameters evaluated showed statistically significant differences between groups in changes at the 6-month follow-up, including dyspareunia (mean [SD] difference, −4.3 [3.4] vs −4.5 [2.3]; *P* = .73), Vaginal Health Index (mean [SD] difference, 3.3 [4.1] vs 5.0 [4.5]; *P* = .17), body image (mean [SD] difference, −3.7 [4.5] vs −2.7 [4.8]; *P* = .35), quality of life (mean [SD] difference, −0.3 [3.6] vs −0.7 [3.2]; *P* = .39). Similarly, there were no differences in improvements in objective outcomes, including vaginal pH (mean [SD] difference, −0.6 [0.9] vs −0.8 [1.2]; *P* = .29), vaginal maturation index (mean [SD] difference, 10.2 [17.4] vs 14.4 [17.1]; *P* = .15), vaginal epithelial thickness (mean [SD] difference, 0.021 [0.014] mm vs 0.013 [0.012] mm; *P* = .30), vaginal epithelial elasticity (mean [SD] difference, −1373 [3197] Pascals vs −2103 [3771] Pascals; *P* = .64).

**Table 3.** Efficacy Outcomes Before and After Treatments by Group

Outcome	Mean (SD)				Difference in change, mean (SD) [95% CI]	*P* value^a^
	Laser (n = 35)		Sham laser (n = 37)			
	Baseline	6 mo	Baseline	6 mo		
**Primary outcome: Female Sexual Function Index score, points** ^b^						
All women)	14.8 (8.8)	20.0 (9.5)	15.6 (7.0)	23.5 (6.5)	2.8 (1.9) [−1.0 to 6.5]	.15
Sexually active women	18.7 (6.1)	21.6 (8.1)	19.0 (4.5)	24.5 (6.5)	2.7 (1.8) [−0.9 to 6.3]	.15
Subscores						
Desire	2.6 (1.2)	3.1 (1.1)	2.2 (0.7)	3.1 (1.0)	NA	NA
Arousal	3.4 (1.5)	4.1 (1.5)	3.7 (1.1)	4.3 (1.3)		
Lubrication	3.3 (1.5)	3.5 (1.6)	3.2 (1.3)	4.1 (1.5)		
Orgasm	3.7 (1.6)	4.0 (1.7)	3.8 (1.5)	4.7 (1.3)		
Satisfaction	3.6 (1.8)	3.8 (2.0)	3.5 (1.7)	4.5 (1.3)		
Pain	2.1 (1.2)	3.3 (1.9)	2.5 (1.3)	3.8 (1.8)		
**Subjective outcomes**						
Dyspareunia^c^	7.3 (2.4)	3.0 (2.8)	7.8 (2.3)	3.3 (2.5)	−0.3 (0.8) [−1.9 to 1.3]	.73
Vaginal Health Index^d^						
Overall	10.8 (3.2)	14.1 (2.9)	10.1 (3.0)	15.1 (4.1)	1.4 (1.0) [−0.6 to 3.5]	.17
Elasticity	2.2 (0.6)	3.1 (0.7)	2.6 (1.2)	3.2 (1.0)	NA	NA
Fluid secretion	2.1 (0.9)	2.9 (0.9)	1.8 (0.8)	3.1 (1.0)		
Vaginal pH	1.3 (0.8)	1.4 (0.8)	1.1 (0.4)	1.7 (1.1)		
Epithelial mucosa	2.6 (0.9)	3.4 (0.8)	2.5 (1.0)	3.5 (0.9)		
Moisture	2.5 (1.0)	3.5 (0.9)	2.1 (0.9)	3.7 (1.0)		
Body Image Scale-Spanish version^e^	12.0 (7.0)	8.3 (6.8)	10.2 (7.1)	7.5 (6.5)	1.1 (1.1) [−1.2 to 3.4]	.35
Short Form 12^f^	31.9 (2.9)	31.6 (3.1)	32.1 (2.3)	31.4 (3.1)	−0.7 (0.9) [−2.5 to 0.9]	.39
**Objective outcomes**						
Vaginal pH	7.7 (0.9)	7.1 (1.0)	7.8 (0.9)	7.0 (1.3)	−0.3 (0.3) [−0.8 to 0.2]	.29
Vaginal Maturation Index, %^g^	7.9 (17.6)	18.1 (19.2)	4.2 (8.8)	18.6 (16.2)	4.3 (4.6) [−4.9 to 13.6]	.15
Vaginal biopsy thickness, mm	0.089 (0.062)	0.110 (0.049)	0.094 (0.060)	0.107 (0.041)	−0.019 (0.018) [−0.05 to 0.017]	.30
Vaginal elasticity, Pascals	4849.8 (2341.9)	3476.5 (1616.5)	5613.8 (3887.8)	3510.5 (1635.2)	−572.2 (1236.6) [−3094.2 to 1949.9]	.64

^a^ *P* values are the mean differences in the variable values of the 2 groups after treatment, assessed with *t* test.

^b^ Range, 2 to 36 points; lower scores indicate worse sexual dysfunction.

^c^ Assessed with a visual analog scale ranging from 0 to 10, with higher score indicating worse dyspareunia.

^d^ Range, 5 to 21; scores of 15 or lower indicate vulvovaginal atrophy.

^e^ Range, 0 to 30; higher scores indicate more concern regarding body image.

^f^ Range, 0 to 100; higher scores indicate better quality of life.

^g^ Range, 0-100; higher scores indicate better vaginal trophism.

No differences were observed between the CLT and SLT groups in terms of adherence to the FLT in the use of ovule moisturizer (mean [SD] uses per month, 9.4 [0.2] vs 9.6 [0.2]; *P* = .61], in the use of the vibrator (mean [SD] uses per month, 5.9 [0.5] vs 5.5 [0.4]; *P* = .45), in monthly sexual relations (mean [SD] events per month, 2.7 [0.4] vs 2.8 [0.4]; *P* = .82), or in attendance to sexual counseling visits (74% vs 83%; *P* = .55)].

### Tolerance and Safety

The mean (SD) tolerance score was 3.3 (1.3) in the CLT group and 4.1 (1.0) in the SLT group (*P* = .007). Complications related and not related to the use of vaginal laser therapy were also recorded, and no differences were identified between groups. Serum estradiol levels were assessed to ensure the safety of the laser in survivors of breast cancer, and no increase from menopausal levels was observed in the CLT group before vs after treatment (mean [SD], 3.1 [5.1] pg/mL vs 3.5 [2.4] pg/mL; *P* = .27). The tolerance and safety of the treatment and the differences between groups are shown in Table 4.

**Table 4.** Tolerance and Safety Assessment Values of the Two Study Groups

Measure	Participants, No. (%)		*P* value
	Laser group (n = 35)	Sham laser group (n = 37)	
Serum estradiol, mean (SD)	6.1 (12.4)	10.7 (3.8)	.27^a^
Tolerance, mean (SD)^b^	3.3 (1.3)	4.1 (1.0)	.007^a^
Related complications, No.			
0	21 (60.0)	28 (75.6)	.67^b^
1	8 (22.8)	6 (16.2)	
2	3 (8.5)	2 (5.4)	
3	2 (5.7)	2 (5.4)	
Severity of related complications^c^			
Mild	16 (45.7)	11 (29.7)	.39^d^
Moderate	4 (11.4)	5 (13.5)	
Severe	0	0	
Nonrelated complications, No.			
0	30 (85.7)	35 (94.6)	.55^d^
1	4 (11.4)	2 (5.4)	
2	1 (2.8)	0	
Severity of nonrelated complications^c^			
Mild	2 (5.7)	1 (2.7)	>.99^d^
Moderate	2 (5.7)	1 (2.7)	
Severe	2 (5.7)	1 (2.7)	

^a^ Assessed with *t* test.

^b^ Tolerance to the intervention was assessed using a Likert scale, with scores ranging from 1 to 5, and with higher scores indicating more tolerability.

^c^ Shown in a CTCAE (Common Terminology Criteria for Adverse Events) scale.

^d^ Assessed with Fisher exact test.

## Discussion

In this RCT including survivors of breast cancer with GSM undergoing treatment with aromatase inhibitors, the subjective and objective outcomes of most participants in both groups improved in symptom severity, sexuality, and vaginal tissue characteristics at the 6-month follow-up . However, there were no differences in the mean improvement between CLT and SLT groups.

Our results suggest that the use of vaginal laser treatment was not effective and was significantly less tolerated than the sham treatment. Nonetheless, since an overall improvement of variables regardless of arm was observed, further studies are needed to determine whether one can attribute overall improvements to the FLT alone or to a placebo effect related to the participating in a trial with an experimental therapy.

The scarcity safe options for sexual dysfunction in survivors of breast cancer^4^ has recently spurred new options of treatment for these women. However, these new treatments still need studies to prove their safety and effecacy.^26,27^ Most studies analyzing vaginal laser treatment efficacy report an improvement, particularly in before vs after studies.^8^ Nevertheless, in the last few years, several RCTs have been published that have challenged this consensus.^28,29,30^ In some RCTs, sexual function improved after the use of vaginal laser treatment compared with placebo^28,29^ and compared with vaginal estrogen treatment.^30^ However, some recent RCTs have questioned these results. Studies by Cruff et al^31^ and Li et al^12^ reported improvement in both SLT and CLT groups for subjective sex-related outcomes and objective outcomes assessing vaginal tissue without significant differences between groups, in keeping with the findings of our study.

Remarkably, vaginal laser treatment appears to be safe, with only mild AEs, such as spotting or vaginal itching, which may be present in approximately 45% of the patients during 5 sessions of treatment. Moderate complications, such as urinary tract infections, were observed in approximately 10% of patients, and no participants reported severe AEs. Moreover, tolerance according to a Likert Scale showed that CLT was a well-tolerated treatment but was significantly worse than SLT.

In this study, many objective outcomes were assessed to provide objectiveness in the evaluation of efficacy. Evaluation of safety and tolerance as well as adherence to treatment were meticulously assessed. Some of the possible biases found in previous RCTs have been taken into consideration in this trial.

### Limitations

This study has some limitations. Our study was limited to survivors of breast cancer undergoing treatment with aromatase inhibitors, which produces ultra-low levels of serum estradiol and may induce a more severe and rapid vaginal atrophy in this subgroup of patients. Therefore, response to the FLT or vaginal laser treatment might be different from that of other populations. This study was performed during the COVID-19 pandemic; therefore, the loss of participants to follow-up was considerable, even though the calculated sample size was achieved. This study did not include a control group without intervention, since it is mandatory to provide FLT to patients presenting symptomatic moderate to severe GSM and to do otherwise would be unethical. Two of the objective outcomes assessed, the VET and VEE, are rarely used in the literature; therefore, further studies are needed to validate their ability to characterize vaginal tissue. Nonetheless, they seem to be promising diagnostic tools for objective evaluation of patients diagnosed with GSM. Additionally, this study described the evaluation of medium-term follow-up (6 months), but further data regarding long-term follow-up is currently being recorded.

## Conclusions

In this RCT, all study participants showed significant improvements with respect to subjective and objective outcomes related to GSM at 6 months’ follow-up, regardless of whether or not they received laser therapy, suggesting that vaginal laser treatment was not effective. Therefore, although vaginal laser treatment was safe, causing often only mild AEs, its efficacy remains to be demonstrated. Further RCTs with a longer follow-up and meta-analysis are needed to confirm the results of this RCT.

## Notes

Supplement 1.
Trial Protocol and Statistical Analysis Plan

Supplement 2. **eFigure.** Percentage of Change of Outcome Measures From Baseline at the 6-Month Follow-up by Group

Supplement 3.
Data Sharing Statement
