# Supplementary material for: Effect of Fractional Carbon Dioxide vs Sham Laser on Sexual Function in Survivors of Breast Cancer Receiving Aromatase Inhibitors for Genitourinary Syndrome of Menopause: The LIGHT Randomized Clinical Trial
Source: JAMA Netw Open. 2023 Feb 10;6(2):e2255697. doi: 10.1001/jamanetworkopen.2022.55697 (PMC9918877; doi:10.1001/jamanetworkopen.2022.55697)
Supplement: Supplement 1. — Trial Protocol and Statistical Analysis Plan [file jamanetwopen-e2255697-s001.pdf]

TRIAL PROTOCOL:

Sexual and Vaginal Health in Breast Cancer Women Receiving Aromatase Inhibitors Before and After CO2 Laser Therapy (LIGHT)

18

19

20

1.1 Promoter Identification

Camil Castelo-Branco/ S Angles Acedo

1.2 Identification of principal investigators of the participating centers

IP: Sonia Angles. Specialist. Gynecology Service (ICGON)

Co-IP: Camil Andreu Castelo-Branco. Senior consultant. Gynecology Service (ICGON)

Collaborating researchers:

- M Immaculate Alonso. Consultant-1. Gynecology Service (ICGON)
- Georgina Casanovas. Statistics-IDIBAPS
- Cristina Marti. Specialist. Gynecology Service (ICGON)
- Eduard Mension. Specialist. Gynecology Service (ICGON)
- Barbara Adamo. Senior Specialist. Medical Oncology (ICMHO)
- Laura Aguilera. Breast Unit Nurse. Gynecology Service (ICGON)
- Angela Arranz. Nursing Cap ICGON
- Gerard Frigola. Resident-3. Pathological Anatomy (CDB)

2. Justification

Sexuality is a central aspect of being human throughout life encompasses sex, gender identities and roles, sexual orientation, eroticism, pleasure, intimacy and reproduction [1]. Most of these aspects may be affected in breast cancer survivors (BCS). In a recent meta-analysis [2, 3] focused on sexual function through Female Sexual Function Index (FSFI) among BCS, female sexual dysfunction prevalence was 73,4% (95 % CI 64–82.8 %, I2 = 96.8 %) and mean FSFI 19.28 (95 % CI 17.39–21.16, I2 = 97.6 %). Several modifiable risk factors for sexual dysfunction in BCS may be optimum targets for intervention: amelioration of vaginal discomfort and urinary incontinence, the benefits of breast conserving surgery on body image or the evaluation of relationship quality [4, 5] Due to the multifaceted nature of the factors affecting sexuality in BCS, most authors [3-10] encouraged the need for comprehensive assessment and for a multidisciplinary approach.

BC diagnosis and/or its treatments (surgery, chemoradiotherapy, hormonal therapy) may altered sexual health. Vulvovaginal health, directly linked to sexual health, is a key factor for female pleasure. Genitourinary syndrome of menopause (GSM) is caused by decline in estrogen at menopause, which may also appear or worsen after systemic cancer treatments as aromatase inhibitors (AI). GSM is associated with sexual symptoms (lack of lubrication, discomfort or pain, impaired function), genital symptoms (dryness, burning, irritation) and urinary symptoms (urgency, dysuria, recurrent urinary tract infections). BCS receiving AI are one of the groups most likely to present severe GSM and sexual complaints [11]. A cross-sectional study [12] on 129 BCS during the first 2 years of adjuvant AI therapy, found 3 out of 4 women were distressed about their sexual problems. Only 52% of women had been sexually active

when endocrine therapy began, but 79% of that group developed new sexual problems.

The first line of treatment for GSM is non-hormonal therapy (regular sexual activity, moisturizers-lubricants, pelvic-floor relaxation techniques, dilators) although in many women these options will not adequately control symptoms. Hormonal therapies must be used with caution in women with estrogen-dependent cancers [13-15]. So, alternative options are emerging for this subset of patients, such as vaginal laser therapy. Different studies [16-18] provided short-term data on non-cancer patients that showed laser therapy was feasible, safe and improved objective and subjective GSM. Data regarding sexuality derived from secondary endpoints and suggested that laser therapy may improve sexual function, mainly decreasing sexual pain [16, 18]. To date, data on laser therapy in BCS is scarce, moreover, few studies included patients receiving ongoing AI [19]. The CO2 laser was the most frequently used device. Different reviews and meta-analysis [20-26] found GSM [27, 28] and sexual function [9, 10] may improve significantly at short-term, however, the body of evidence is of low quality. Therefore, before recommendation for the use of laser therapy for sexual complaints in BCS with GSM, there are unmet needs to be solved as efficacy and safety at long-term, biases related to patients' expectations of therapy and a detailed assessment of the complex underpinnings of sexuality.

3. Objectives

To answer the unmet needs previously mentioned, the aim of the current study is to verify the outcomes of sexual and vaginal health in breast cancer women receiving AI, who were experiencing symptoms of GSM, before and after CO2 laser therapy compared to a sham-controlled group.

Specifically, we will compare the following subjective and objective measurements of sexual and vaginal health:

MAIN GOAL: Report an improvement in sexuality:

- Primary outcome: sexual function (FSFI total score)
- Secondary sexual outcomes: resumption sexual activity (sexually active vs non-sexually active), sexual activity frequency (nº sexual activity/week), dyspareunia (VAS), female sexual dysfunction (VAS sexual life distress), sexual dimensions: desire, arousal, lubrication, orgasm, satisfaction and pain (FSFI) and body image (S-BIS),.

OTHER OBJECTIVES: Report an improvement on GSM symptoms (efficacy) and quality of life, feasibility and safety of laser therapy in BCS:

- Secondary non-sexual outcomes: quality of life (SF-12). Verify an acidification of the vaginal pH, an improvement in the maturation index, improvement in the Gloria Backmann Index: Vaginal Health Index and pathologic evaluation of vaginal tissue samples. To evaluate toxicity associated with vaginal laser therapy in this population (AEs and SAEs). To determine how many women with the defined patient eligibility will complete all treatments.

4. Study Description

Background: Vulvovaginal health, directly linked to sexual health, is a key factor for female pleasure. BCS receiving AI are likely to present severe GSM and sexual complaints. Innovative options, as vaginal laser therapy, are emerging to treat GSM and sexual dysfunctions. Nowadays, data in BCS is scarce, moreover, few studies included patients receiving AI. Different meta-analysis found GSM and sexual function may improve significantly at short-term, however, the body of evidence is of low quality. Therefore, before recommendation of laser therapy for sexual complaints in BCS with GSM, there are unmet needs to be solved: efficacy and safety at long-term, biases related to patients' expectations and a detailed assessment of the complex underpinnings of sexuality.

Aims: To evaluate sexual and vaginal health in BCS receiving AI with GSM, before and after CO2 laser therapy compared to a sham-controlled group.

Methods: Prospective, randomized, double-blind controlled study with two parallel study arms: 1) Fractional CO2 laser therapy (5monthly sessions). 2) Sham laser therapy (5monthly sessions). After end-treatment, patients are followed up at 1 month and 6 months. BCS treated or undergoing AI with GSM and sexual function impairment, will be suitable. All patients will maintain first-line non-hormonal treatment and sexual assessment (PLISSIT Model) according with usual care. The primary outcome is improvement in sexual function (FSFI total score). As secondary outcomes: resumption

sexual activity, sexual activity frequency, dyspareunia (VAS), female sexual dysfunction, sexual dimensions (FSFI), body image (S-BIS), quality of life (SF-12), vaginal pH acidification, maturation index and Vaginal Health Index of Gloria Backmann, adverse events, satisfaction (Likert scale) and adherence to treatment.

Expected impact: Emergent, non-invasive, laser therapy has significant benefit for BCS with AI, improving subjective and objective sexual and vaginal health outcomes and adding value to the usual care multidisciplinary approach.

5. Study Design

Study Type: Interventional (Clinical Trial)

Actual Enrollment: 84 participants

Allocation: Randomized

Intervention Model: Parallel Assignment

Intervention Model Description: Randomized, double-blind, sham-controlled trial

Masking: Quadruple (Participant, Care Provider, Investigator, Outcomes Assessor)

Primary Purpose: Treatment

6. Arms

1) Active Comparator: EFFECTIVE LASER (CLT)

Arm using basal treatment and adding vaginal laser using double blind to adjust the treatment to regular potence.

Device: CO2 LASER 5 SESSIONS OF CO2 VAGINAL LASER

2) Sham Comparator: SHAM LASER (SLT)

Arm using basal treatment and adding vaginal laser using double blind to adjust the treatment to zero potence.

Device: CO2 SHAM LASER 5 SESSIONS OF CO2 VAGINAL SHAM LASER

7. Interventions

At the first visit the patients will fill in all the questionnaires, and a vaginal examination will be performed to evaluate the genital tract and collect samples for analysis, as described in section Outcomes.

1. First line therapy (FLT)

All patients from both arms are instructed to use the FLT, which is supplied to every participant during the study. This therapy consists of a hormone-free moisturizer containing hyaluronic acid used every 3 days, a daily external vaginal hormone-free moisturizer, and a vaginal vibrator to be used two times per week for 5-10 minutes each with the help of intimate lubricant. A personal calendar is given to each patient in which they record every use of the moisturizer, the vaginal vibrator, and each sexual relation practiced. Additionally, specialized sexual assessment is also offered as an optional visit to all the participants of the study. This includes a sexual interview to assess their past and current sexual life, as well as future sexual expectations. Women

who attend this visit receive sexual counseling based on the permission, limited information, specific suggestions and intensive therapy (PLISSIT) model.

2. Preparation for the procedure

The patients are cited between 4 to 6 weeks after the first visit. They are instructed to avoid intercourse and use an internal vaginal ovule moisturizer daily five days prior and five days after the laser session, and use topic lidocaine cream 1 hour before the laser session.

3. Laser treatment

All patients receive five sessions one month apart from the vaginal laser treatment. The treatment is performed by a professional blinded to the treatment group.

CLT is performed using the fractional microablative CO2 laser system, SmartXide2 V^2LR, MonaLisa Touch^{TM}, (DEKA Laser, Calenzano, FI, Italy) at standard settings (40W power, 1000 µs dwell time, 1000 µm DOT spacing, SmartStack 2 on Double Pulse emission mode), with a delivery fluence of 5.37 J/cm2.

SLT was performed at minimal energy settings to avoid any tissue effect (0.0 W power, 100 µs dwell time, 2000 µm DOT spacing, SmartStack 1 on SmartPulse emission mode), delivering no energy (fluence, 0 J/cm2).

All patients complaining of symptoms suspicious of vulvovaginal candidiasis or urine infection prior to the laser session are treated accordingly, and the session is re-scheduled.

The first step of the procedure involves removal of the external anesthetic cream with a dry gauze. Then, using an exploration speculum, a new dry gauze is inserted into the

vaginal canal to remove all residual vaginal moisture. Next, the laser probe is inserted into the vagina without lubrication. A 360º laser probe is used as the first option, but when the diameter is too large, a 90º probe will be used. Then, the laser pulses are delivered to treat the entire circumference and length of the vagina from the apex to the introitus. Patients have no visual stimuli since opaque glasses are used; neither olfactory stimulus from smoke plume due to the use of an aspirator during the procedure. Auditory stimuli from the laser and aspirator are set to be equal between the two groups.

Following the laser session, a questionnaire of tolerance of each session, vaginal complaints and secondary effects is completed.

8. Outcomes

Outcomes are obtained on the first visit prior to the initiation of any treatment and six months later (i.e., 1 month after the 5th laser session).

1. Female Sexual Function Index (FSFI)

FSFI is a generic sexual questionnaire, also validated for cancer survivors. It assesses six sexual dimensions (desire, arousal, lubrication, orgasm, satisfaction, and pain). Global sexual function results in a score ranging from 2 to 36, with a higher score indicating better sexual function. A cut-off ≤ 26.55 identifies women at risk of female sexual dysfunction. The FSFI was analyzed in all the patients included irrespective of initial sexual activity status.

2. Visual Analog Scale (VAS) of dyspareunia

The intensity of dyspareunia is also assessed in all patients (sexually active and inactive) at the baseline visit according to their last vaginal sexual activities. Patients are asked to fill in a VAS ranging from 0 to 10.

3. Spanish- Body Image Scale Test (S-BIS test)

The S-BIS, is a Spanish validated questionnaire assessing affective, behavioral, and cognitive body image dimensions in 10 items. The total score is the sum of all the items (range 0-30), with higher scores indicating more concern regarding body image.

4. Quality of Life Short Form 12 (SF-12 test)

SF-12 consists of a total of 12 items in 8 subdimensions on physical functioning. Scores range from 0 to 100, with higher scores indicating better health status.

5. Vaginal pH

To assess vaginal pH, a piece of litmus paper is placed on the lateral vaginal wall until moistened. A pH of 4.6 or higher indicates vaginal atrophy.

6. Vaginal Health Index (VHI)

The VHI subjectively assesses the elasticity of the vagina, the amount of discharge, the integrity of the epithelium and humidity, along with pH as the only objective criteria. The results range from 5 to 21 and scores ≤15 indicate vulvovaginal atrophy.

7. Vaginal Maturation Index

Cytological samples are assessed by gynecological cytologists blinded to the randomization group and sample sequence (before or after treatment). The relative proportion of parabasal, intermediate, and superficial vaginal epithelial cells is assessed.

8 Vaginal Epithelium Thickness (VET)

Two full-thickness vaginal mucosal samples are taken from the right vaginal wall 2-3 cm above the introitus were obtained using Tischler biopsy forceps after local lidocaine infiltration. One of the specimens is fixed in formalin and routinely embedded in paraffin for histological evaluation. Four µm sections is stained with hematoxylin and eosin and digitized using a IntelliSite Ultra-Fast Scanner (Philips, Eindhoven, Netherlands). The slides are evaluated and measured by a gynecologic pathologist. VET is microscopically evaluated averaging the three areas showing the maximum VET and the three areas demonstrating the minimum VET in hematoxylin and eosin-stained tissue samples.

9. Vaginal Epithelium Elasticity (VEE)

The second biopsy sample is used for evaluation of elasticity. VEE measurements are conducted using a customized Atomic Force Microscope (TE2000; Nikon, Tokyo, Japan) equipped with a V-shape cantilever (0.13 N/m, nominal spring constant K) ending with a polystyrene bead spherically shaped with a radius of 4.5 µm (Novascan technologies, Ames, IA, USA). Micromechanics are examined by indenting the sample with the bead while recording the force applied. The biophysics investigators are blinded to the randomization group and sample sequence.

10. Adverse Effects

Adverse effects are evaluated after every laser session and recorded and graded according to the National Cancer Institute Common Terminology Criteria for Adverse Events v5.0.

9. Eligibility Criteria

The study will be conducted in the Breast Cancer Unit of the Hospital Clinic of Barcelona, Spain. The inclusion criteria are: 1) BCS patients aged 30 years and older receiving AI; 2) menopause, signs/symptoms of GSM with dyspareunia and vaginal pH ≥5; and 3) willingness to have sex. The exclusion criteria include: a) use of vaginal moisturizers and/or lubricants in the last 30 days; b) vaginal hormonal treatment in the last 6 months; c) use of radiofrequency, laser treatment, hyaluronic acid or lipofilling in the vagina in the last two years; d) ospemifene treatment; e) intraepithelial neoplasm of cervix, vagina, or vulva; f) active genital tract infection; g) prior treatment for genital

cancer; h) organ prolapse stage ≥II on pelvic examination (POP-Q); and i) positive test result for human papillomavirus.

Written informed consent will be obtained from all the participants.

10. Statistical analysis and Blinding

Considering the FSFI score as the main study variable, a sample size of 33 women is calculated for each group, accepting an alpha risk=0.05 and a beta risk <0.1 in a bilateral contrast. The common standard deviation is considered to be of 5 points and the minimum expected effect size is 4 points. Assuming a loss to follow-up of 15%, the calculated sample size is 76 patients.

Participants will be equally assigned by 1:1 block randomization to either CLT or SLT using the STATA software, version 15.1 (StataCorp LLC, College Station, TX, USA). The block sizes are eight. Allocation concealment will be performed using a protected personal code folder on the hospital intranet. Access to the randomization folder is limited to an authorized collaborator physician who has no other involvement in the study.

Statistical analyses will be performed with the Software 15.1 STATA, v. 15.1). A descriptive analysis of all data will be performed. Normal distribution of the sample will be evaluated using the Shapiro-Wilk test. Continuous variables will be compared using the independent or paired-samples T-test and presented as mean ± standard

deviation. Contingency tables will be assessed using the Fisher exact test. A $p<0.05$ will be considered statistically significant.

The laser parameters will be manually inserted by an assistant and remained blind to the gynecologist and participants. Only the assistant will have access to the randomization folder. The patients would not be able to guess in which group they are allocated, as they will be informed that the laser treatments might not produce any discomfort.

11. Expected impact

The BCS receiving AI are at higher risk of developing GSM symptoms. Sexual issues are common, either secondary to painful sex or as a direct effect of estrogen deprivation in the brain. However, addressing sexual health issues in BCS still meets several barriers from both, patient and health care professionals. GSM symptoms do not put survival at risk, however, have a significant impact in the quality of life [26].

The first-line treatment for GSM symptoms should be non-hormonal therapies according clinical guidelines, although frequently it will be insufficient to alleviate symptoms. Hormonal therapies are not currently recommended and must be used with caution in BCS [13-15]. So, energy-based treatments have emerged as a promising option in this subgroup of patients. However, the body of evidence is lacking to make decisive recommendations [18], specially to treat sexual complaints. There is an urgent need to carry out RCT with larger sample size, long-term follow-up and blinded control

group for answer various unmet needs in this field: short/mid and long-term safety issues and efficacy, treatment modalities (type of laser) and protocols (laser parameters, sessions numbers, repetitions).

This project aims to demonstrate that emergent, non-invasive, non-anesthetic laser therapy has significant benefit for BCS with AI, measured in a prospective, randomized, double-blind controlled trial using validated tools for subjective and objective sexual and vaginal health outcomes and quality of life.

Moreover, sexual health is a state of physical, emotional, mental and social well-being in relation to sexuality which requires the possibility of having pleasurable and safe sexual experiences [1]. So, both arms of study groups will benefit from multidisciplinary approach including non-hormonal therapies (regular sexual activity, moisturizers, lubricants, pelvic floor relaxation techniques and/or dilators) and sexual assessment using the PLISSIT Model according with usual care in our hospital and with international recommendations [7, 8, 18].

---

12. References

1. Palacios S, Castelo-Branco C, Currie H, Mijatovic V, Nappi RE, Simon J, Rees M. Update on management of genitourinary syndrome of menopause: A practical guide. Maturitas 2015;82(3):308-13.

2. Palacios S, Cancelo MJ, Castelo Branco C, Llaneza P, Molero F, Borrego RS. Vulvar and vaginal atrophy as viewed by the Spanish REVIVE participants: symptoms, management and

353 treatment perceptions. Climacteric. 2017 Feb;20(1):55-61.

354 3. Castelo-Branco C, Biglia N, Nappi RE, Schwenkhagen A, Palacios S. Characteristics of
355 post-menopausal women with genitourinary syndrome of menopause: Implications for
356 vulvovaginal atrophy diagnosis and treatment selection.

357 Maturitas. 2015;81(4):462-9.

358 4. Naumova I, Castelo-Branco C. Current treatment options for postmenopausal vaginal
359 atrophy. Int J Womens Health. 2018; 10:387-395.

360 5. Sanchez-Borrego R, Molero F, Castaño R, Castelo-Branco C, Honrado M, Jurado AR et
361 al. Spanish consensus on sexual health in men and women over 50. Maturitas. 2014;78(2):138-
362 45.

363 6. Nappi RE, Panay N, Bruyniks N, Castelo-Branco C, De Villiers TJ, Simon JA. The clinical
364 relevance of the effect of ospemifene on symptoms of vulvar and vaginal atrophy. Climacteric.
365 2015 Apr;18(2):233-40.

366 7. Bruyniks N, Nappi RE, Castelo-Branco C, de Villiers TJ, Simon J. Effect of ospemifene on
367 moderate or severe symptoms of vulvar and vaginal atrophy. Climacteric. 2016;19(1):60-5.

368 8. Del Pup L. Ospemifene: a safe treatment of vaginal atrophy. Eur Rev Med Pharmacol
369 Sci 2016; 20:3934–3944

370 9. Lester J, Pahouja G, Andersen B, et al. Atrophic vaginitis in breast cancer survivors: a
371 difficult survivorship issue. J Pers Med 2015;5:50–66

372 10. Fallowfield L, Cella D, Cuzick J, et al. Quality of life of postmenopausal women in the
373 Arimidex, Tamoxifen, Alone or in Combination (ATAC) Adjuvant Breast Cancer Trial. J Clin
374 Oncol 2004;22:4261–71

11. Cella D, Fallowfield L, Barker P, et al. Quality of life of postmenopausal women in the ATAC (''Arimidex'', tamoxifen, alone or in combination) trial after completion of 5 years' adjuvant treatment for early breast cancer. Breast Cancer Res Treat 2006;100:273–84.

12. Schover LR, Baum GP, Fuson LA, Brewster A, Melhem-Bertrandt A (2014) Sexual problems during the first 2 years of adjuvant treatment with aromatase inhibitors. J Sex Med 11(12):3102–3111.

13. Greendale G, Petersen L, Zibecchi L, Ganz P. Factors related to sexual function in postmenopausal women with a history of breast cancer. Menopause. Vol. 8, No. 2, pp. 111–119

14. Boquiren VM, Esplen MJ, Wong J, Toner B, Warner E, Malik N. Sexual functioning in breast cancer survivors experiencing body image disturbance. Psychooncology. 2016;25(1):66-76.

15. Jing L, Zhang C, Li W, Jin F, Wang A. Incidence and severity of sexual dysfunction among women with breast cancer: a meta-analysis based on female sexual function index. Supportive Care in Cancer (2019) 27:1171–1180.

16. Fatehi S, Maasoumi R, Atashsokhan G, Hamidzadeh A, Janbabaei G, Mirrezaie SM. The effects of psychosexual counseling on sexual quality of life and function in Iranian breast cancer survivors: a randomized controlled trial. Breast Cancer Res Treat. 2019;175(1):171-179.

17. Faghani S, Ghaffari F. Effects of Sexual Rehabilitation Using the PLISSIT Model on Quality of Sexual Life and Sexual Functioning in Post-Mastectomy Breast Cancer Survivors. Asian Pac J Cancer Prev. 2016;17(11):4845-4851. Published 2016 Nov 1.

18. Athanasiou S, Pitsouni E, Douskos A, Salvatore S, Loutradis D, Grigoriadis T. Intravaginal energy-based devices and sexual health of female cancer survivors: a systematic review and meta-analysis. Lasers Med Sci. 2020;35(1):1-11.

19. Jha S, Wyld L, Krishnaswamy PH. The Impact of Vaginal Laser Treatment for Genitourinary Syndrome of Menopause in Breast Cancer Survivors: A Systematic Review and Meta-analysis. Clin Breast Cancer. 2019;19(4):e556-e562.

20. Sánchez-Borrego R, Mendoza N, Beltrán E, Comino R, Allué J, Castelo-Branco C et al. Position ofthe Spanish Menopause Society regardingthe management of menopausal symptoms in breast cancer patients. Maturitas.2013 Jul;75(3):294-300.

21. Oyarzun MFG,Castelo-Branco C. Local hormone therapy for genitourinary syndrome of menopause in breast cancerpatients: is it safe? Gynecol Endocrinol. 2017;33(6):418-420.

22. Santen RJ. Vaginal administration of estradiol: effects of dose, preparation and timing on plasma estradiol levels. Climacteric 2015; 18:121–34

23. Le Ray I, Dell'Aniello S, Bonnetain F, et al. Local estrogen therapy and risk of breast cancer recurrence among hormone-treated patients: a nested case-control study. Breast Cancer Res Treat 2012;135:603–9

24. Santos I, Clissold S. Urogenital disorders associated with oestrogen deficiency: the role of promestriene as topical oestrogen therapy. Gynecol Endocrinol 2010;26:644–51

25. Del Pup L, Di Francia R, Cavaliere C, et al. Promestriene, a specific topic estrogen. Review of 40 years of vaginal atrophy treatment: is it safe even in cancer patients? Anticancer Drugs 2013;24:989–98

26. Kendall A, Dowsett M, Folkerd E et al. Caution: vaginal estradiol appears to be contraindicated in postmenopausal women on adjuvant aromatase inhibitors. Ann Oncol 2006;17:584–7

27. Wills S, Ravipati A, Venuturumilli P, et al. Effects of vaginal estrogens on serum estradiol levels in postmenopausal breast cancer survivors and women at risk of breast cancer

taking an aromatase inhibitor or a selective estrogen receptor modulator. J Oncol Pract 2012;8:144–8

28. Goldstein SR, Bachmann GA, Koninckx PR, et al. Ospemifene 12-month safety and efficacy in postmenopausal women with vulvar and vaginal atrophy. Climacteric 2014;17:173–82

29. Salvatore S, Athanasiou S, Candiani M. The use of pulsed CO2 lasers for the treatment of vulvovaginal atrophy. Curr Opin Obstet Gyneco 2015; 6:504-8

30. Tien YW, Hsiao SM, Lee CN, Lin HH. Effects of laser procedure for female urodynamic stress incontinence on pad weight, urodynamics, and sexual function. Int Urogynecol J 2016

31. Tadir Y, Gaspar A, Lev-Sagie A et al. Light and energy based therapeutics for genitourinary syndrome of menopause: consensus and controversies. Lasers Surg Med 2017;49:137–159

32. Pardo JL, Sola VR, Morales AA. Treatment of female stress urinary incontinence with Erbium-YAG laser in non-ablative mode. Eur J Obstet Gynecol Reprod Biol 2016;204:1-4

33. Salvatore S, Pitsouni E, Del Deo F, Parma M, Athanasiou S, Candiani M. Sexual Function in Women Suffering From Genitourinary Syndrome of Menopause Treated With Fractionated CO2 Laser. Sex Med Rev. 2017;5(4):486-494.

34. Perino A, Calligaro A, Forlani F, et al. Vulvovaginal atrophy: a new treatment modality using thermos-ablative fractional CO2 laser. Maturitas 2015;80:296–301

35. Salvatore S, Nappi RE, Zerbinati N, et al. A 12-week treatment with fractional CO2 laser for vulvovaginal atrophy: a pilot study. Climacteric 2014;17:363–369

36. Pieralli A, Bianchi C, Longinotti M, et al. Long-term reliability of fractioned CO2 laser as

443 a treatment for vulvovaginalatrophy (VVA) symptoms. Arch Gynecol Obstet. 2017
444 Nov;296(5):973-97

445 37. Quick AM, Zvinovski F, Hudson C, et al. Fractional CO2 laser therapy for genitourinary
446 syndrome of menopause for breast cancer survivors. Support Care Cancer. 2020;28(8):3669-
447
