# Supplementary material for: Effect of Fractional Carbon Dioxide vs Sham Laser on Sexual Function in Survivors of Breast Cancer Receiving Aromatase Inhibitors for Genitourinary Syndrome of Menopause: The LIGHT Randomized Clinical Trial
Source: JAMA Netw Open. 2023 Feb 10;6(2):e2255697. doi: 10.1001/jamanetworkopen.2022.55697 (PMC9918877; doi:10.1001/jamanetworkopen.2022.55697)
Supplement: Supplement 2. — eFigure. Percentage of Change of Outcome Measures From Baseline at the 6-Month Follow-up by Group [file jamanetwopen-e2255697-s002.pdf]

# Supplemental Online Content

Mension E, Alonso I, Anglès-Acedo S, et al. Effect of fractional carbon dioxide vs sham laser on sexual function in survivors of breast cancer receiving aromatase inhibitors for genitourinary syndrome of menopause: the LIGHT randomized clinical trial. *JAMA Netw Open.* 2023;6(2):e2255697. doi:10.1001/jamanetworkopen.2022.55697

**eFigure.** Percentage of Change of Outcome Measures From Baseline at the 6-Month Follow-up by Group

This supplemental material has been provided by the authors to give readers additional information about their work.

© 2023 Mension E et al. *JAMA Network Open*.

**eFigure.** Percentage of Change of Outcome Measures From Baseline at the 6-Month Follow-up by Group

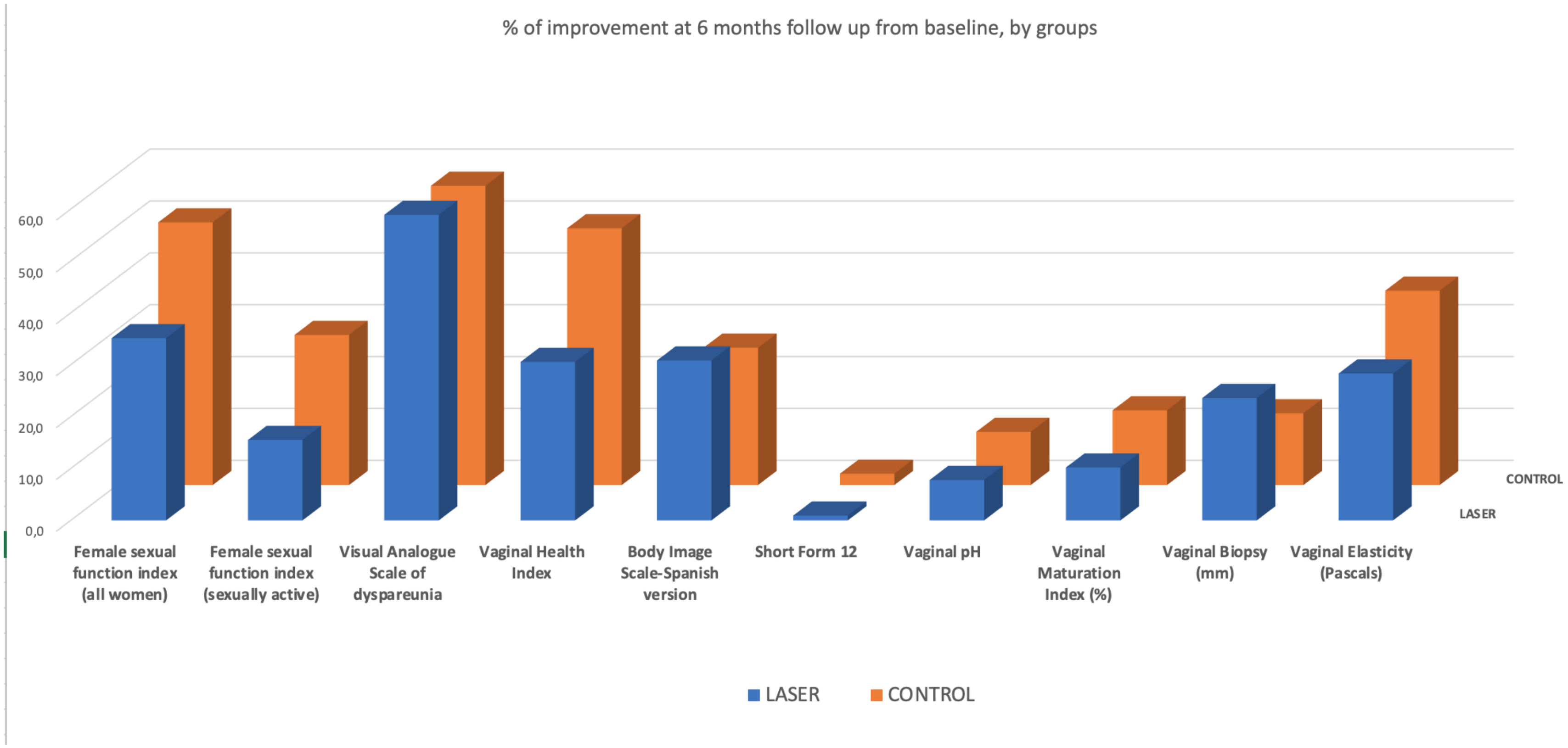


© 2023 Mension E et al. *JAMA Network Open*.
