# Supplementary material for: Effect of Fractional Carbon Dioxide vs Sham Laser on Sexual Function in Survivors of Breast Cancer Receiving Aromatase Inhibitors for Genitourinary Syndrome of Menopause: The LIGHT Randomized Clinical Trial
Source: JAMA Netw Open. 2023 Feb 10;6(2):e2255697. doi: 10.1001/jamanetworkopen.2022.55697 (PMC9918877; doi:10.1001/jamanetworkopen.2022.55697)
Supplement: Supplement 3. — Data Sharing Statement [file jamanetwopen-e2255697-s003.pdf]

Data Sharing Statement

Mension. Effect of Fractional Carbon Dioxide vs Sham Laser on Sexual Function in Survivors of Breast Cancer Receiving Aromatase Inhibitors for Genitourinary Syndrome of Menopause. *JAMA Netw Open*. Published February 10, 2023. doi:10.1001/jamanetworkopen.2022.55697

Data
Data available: Yes
Data types: Deidentified participant data
How to access data: request for data must be sent to an individual: mension@clinic.cat
When available: With publication

Supporting Documents
Document types: None

Additional Information
Who can access the data: Researchers whose proposed use of the data has been approved
Types of analyses: for a specified purpose
Mechanisms of data availability: after approval of a proposal with a signed data access agreement
